# Development of Level A In Vitro–Vivo Correlation for Electrosprayed Microspheres Containing Leuprolide: Physicochemical, Pharmacokinetic, and Pharmacodynamic Evaluation

**DOI:** 10.3390/pharmaceutics12010036

**Published:** 2020-01-02

**Authors:** Dong-Seok Lee, Dong Wook Kang, Go-Wun Choi, Han-Gon Choi, Hea-Young Cho

**Affiliations:** 1 College of Pharmacy, CHA University, 335 Pangyo-ro, Bundang-gu, Seongnam-si, Gyeonggi-do 13488, Korea; movingstone0620@gmail.com (D.-S.L.); dongwk203@gmail.com (D.W.K.); gwchoi153@gmail.com (G.-W.C.); 2 College of Pharmacy & Institute of Pharmaceutical Science and Technology, Hanyang University, 55 Hanyangdaehak-ro, Sangnok-gu, Ansan 426-791, Korea; hangon@hanyang.ac.kr

**Keywords:** in vitro–in vivo correlation, leuprolide, microspheres, sustained release, pharmacokinetics, pharmacodynamics

## Abstract

This study optimized the preparation of electrosprayed microspheres containing leuprolide and developed an in vitro–in vivo correlation (IVIVC) model that enables mutual prediction between in vitro and in vivo dissolution. The pharmacokinetic (PK) and pharmacodynamic (PD) study of leuprolide was carried out in normal rats after subcutaneous administration of electrosprayed microspheres. The parameters of the IVIVC model were estimated by fitting the PK profile of Lucrin depot^®^ to the release compartment of the IVIVC model, thus the in vivo dissolution was predicted from the in vitro dissolution. From this correlation, the PK profile of leuprolide was predicted from the results of in vivo dissolution. The IVIVC model was validated by estimating percent prediction error (%PE) values. Among prepared microspheres, an optimal formulation was selected using the IVIVC model. The maximum plasma concentration and the area under the plasma concentration–time curve from zero to infinity from the predicted PK profile were 4.01 ng/mL and 52.52 h·ng/mL, respectively, and from the observed PK profile were 4.14 ng/mL and 56.95 h·ng/mL, respectively. The percent prediction error values of all parameters did not exceed 15%, thus the IVIVC model satisfies the validation criteria of the Food and Drug Administration (FDA) guidance. The PK/PD evaluation suggests that the efficacy of OL5 is similar to Lucrin depot^®^, but the formulation was improved by reducing the initial burst release.

## 1. Introduction

Sustained release (SR) injections containing poly (d,l-lactide-co-glycolide) (PLGA) and its copolymers have been widely used because of their biocompatibility and their ability to achieve a variety of therapeutic benefits, including no need for daily administration, more consistent bioavailability, low relapse rates, and improved patient compliance. In addition, there are several marketed products based on PLGA, including triptorelin, leuprolide, goserelin, risperidone, naltrexone, doxycycline, octreotide, and growth hormone [1]. Among these, leuprolide, goserelin, octreotide, growth hormone, and lanreotide are notable for being peptide drugs.

The leuprolide used in this study is a synthetic nonapeptide gonadotropin-releasing hormone (GnRH) analog that acts as a potent reversible inhibitor of gonadotropin secretion through suppression of testicular steroidogenesis. Continuous administration of the leuprolide results in reduced testosterone levels through negative feedback. These properties make it useful for various hormone therapies such as prostate cancer, endometriosis, and central precocious puberty, among others. However, as a peptide drug alone, it is easily broken down by plasma peptidase, which makes it challenging to maintain therapeutic drug concentrations for any duration.

The SR technologies used for leuprolide have been commonly employed in the pharmaceutical industry. The water-in-oil-in-water (w/o/w) emulsion is generally used for encapsulating a water-soluble drug such as a peptide or a protein. Lupron depot, which was developed by Takeda in 1989, was approved by the FDA and is currently available as an SR formulation of leuprolide. In this method, after the aqueous solution containing the drug is dispersed in an organic solvent containing the polymer, the water-soluble drug is dispersed in an aqueous phase surrounded by an oil phase. This method has the disadvantage that the yield and the encapsulation rate of the drug are low, because a large amount of the drug escapes during the process of drug dispersal in the water phase. In addition, initial release rates of the drug are relatively high since the microsphere porosity is increased compared to other manufacturing methods. The standard method of manufacturing SR injections is spray-drying, in which the solutions containing drug and polymer are spray-dried by contact with hot circulating air. This method has a short formulation time and a yield of nearly 100% [2]. Additionally, spray-drying is a single-step process, and features such as particle size can easily be controlled by changing process parameters; however, there is a potential risk of denaturing peptide drugs [3]. In this study, the electrospray method was used to prepare the sustained release formulation. This technology is a one-step process that has a short manufacturing time and does not need hot air at all. It enables the acquisition of a high yield, provides easy control of drug particle size, and reduces the risk of heat-induced denaturation of the peptide drugs [4,5]. Despite these advantages, formulation study of leuprolide using the electrospray method has never been reported. Therefore, this manuscript is the first study on microsphere formulation of leuprolide using the electrospray method.

For development and optimal selection of formulations, numerous formulation studies and clinical trials are required for a long period. The introduction of an in vitro–in vivo correlation (IVIVC) for the development of SR formulation could greatly reduce these efforts. 

IVIVC is a mathematical model that finds a correlation between results of in vitro dissolution kinetics and in vivo plasma concentration of a drug. IVIVC is classified into four categories (Level A, Level B, Level C, and Multiple Level C) according to data type, prediction range, and prediction power. Among them, Level A correlations are the most common type developed in new drug applications (NDAs) submitted to the Food and Drug Administration (FDA). Level A correlations are generally estimated by a two-stage method: deconvolution and then comparison of the fraction of drug absorbed to the fraction of drug dissolved. This relationship is commonly linear and shows a point-to-point correlation between in vitro dissolution and in vivo input rate of the drug from the dosage form. Several IVIVC studies on SR injection formulations have been conducted. D’Souza et al. introduced IVIVC on long acting microspheres of olanzapine [6]. They established Level A IVIVC through in vitro release data obtained from a dialysis-based method using deconvolution and fractional area under the plasma concentration-time curve (AUC) method. Regarding SR injections of risperidone, Shen et al. and Hu et al. both conducted IVIVC studies [7,8]. Shen et al. obtained in vitro results using United States Pharmacopeia (USP) apparatus 4 for formulations of risperidone prepared through several different processes and developed Level A IVIVC using the Loo-Riegelman method. Hu et al., meanwhile, studied the in vitro release conditions including pH, osmotic pressure, and temperature, which best reflect an IVIVC of risperidone. Most IVIVC studies have established the correlation by traditional deconvolution methods such as the Wagner-Nelson and the Loo-Riegelman methods. However, the deconvolution method has difficulties in determining the correlation between in vitro and in vivo profiles of SR injection, which makes it difficult to interpret its disposition as a conventional one-compartment or two-compartment model. Li et al. established an IVIVC model for exenatide through simulation modeling to overcome the shortcomings of the traditional deconvolution method [9]. The simulation model they developed is more predictable than the deconvolution method when using the same in vitro and in vivo data. However, their study was retrospective and used results obtained following pharmacokinetic (PK) experiments with several doses of the same formulation. This makes it difficult to use as a tool to predict PK during the early stages of formulation studies.

In this study, the leuprolide and PLGA were formulated into microspheres using the electrospray method. Preparation of microspheres was optimized by varying solvents and the mixing ratio of two types of PLGA, RG502 and RG502H. Then, the physicochemical properties of the electrosprayed microspheres were assessed. The drug contents of microspheres were calculated, and the dissolution tests were performed. In addition, we developed an IVIVC model as a tool that can be used in the early stages of drug development. While most traditional IVIVCs comprise retrospective research based on in vivo results, this study can predict the PK profile during the formulation study using in vitro results with minimal in vivo results. For this reason, this paper is expected to provide a method both financially and timely efficient to the pharmaceutical industry. The model was validated by calculating the PE values between the predicted values from IVIVC and the observed values from PK results of the trial formulation. Based on this, PK profiles of trial formulations were predicted, and one formulation that had the most similar PK pattern to the reference drug (Lucrin depot^®^) was selected. Then, PK and pharmacodynamics (PD) of the leuprolide after subcutaneous (SC) administration of trial formulation was evaluated.

## 2. Materials and Methods 

### 2.1. Chemicals

The reference standards of leuprolide acetate (purity > 98%) and leuprolide-d5 acetate (purity > 97%) as internal standard (IS) were purchased from Toronto Research Chemicals (Toronto, ON, Canada). Poly (d,l-lactide-co-glycolide) (PLGA), acetic acid, and Tween 80 was purchased from Sigma-Aldrich (St. Louis, MO, USA) including both RG502 and RG502H. Lucrin depot^®^ (leuprolide acetate, 3.75 mg) was purchased from Abbvie Korea (Seoul, Korea). Acetonitrile, acetone, ethanol, methanol, ethyl acetate, and methylene chloride were purchased from J.T. Baker (Phillipsburg, NJ, USA). HPLC grade water was obtained from an ElgaPurelab Option-Q system (ElgaLabWater, Marlow, UK) and was used throughout this study. The other chemicals were of HPLC grade or better.

### 2.2. Instruments

ESR 100 electrospraying equipment (NanoNC, Seoul, Korea) and gas-tight syringes (Hamilton Co, Reno, NV, USA) were used for the preparation of microspheres. SEM (S-4800; Hitachi Ltd., Tokyo, Japan), differential scanning calorimetry (DSC) (DSC Q20; TA Instruments, New Castle, DE, USA), FTIR spectrophotometer-430 (Shimadzu Corporation, Tokyo, Japan), sputter coater (K575K; EMI Teck, UK), and heating dry bath (confido–S20H, Seoul, Korea) were used for the physicochemical evaluation. The HPLC system for release test and drug content assay consisted of an Alliance^Ⓡ^HPLC e2695 system with 2489 UV/Vis Detector. The UPLC-MS/MS system for bioanalysis consisted of Acquity^™^UPLC system (Waters Corp., Milford, MA, USA) coupled with a Mass Spectrometer (Xevo™ TQ-S, Waters Corp., Milford, MA, USA). The testosterone ELISA kit (R&D Systems, Minneapolis, MN, USA) was used for determination of testosterone in rat plasma.

### 2.3. Animals

Fifteen male Wistar rats were obtained from Dae Han Biolink (Eumseong, ChungCheongbook-do, Korea). All the rats were maintained on a 12 h dark–light cycle at ambient temperature (23 ± 2 °C) and relative humidity (50 ± 5%) with free access to water and food. This study was conducted according to the Guidelines for Ethical Conduct in the Care and Use of Animals and the rules of Good Laboratory Practice (American Psychological Association, 1992) and was approved by the Institutional Animal Care and Use Committee (IACUC, No. 150035, approval date: 10 April 2015) at CHA laboratory animal research center. The rats were fasted for approximately 12 h with free access to water.

### 2.4. Preparation of Electrosprayed Microspheres

**Figure 1:**
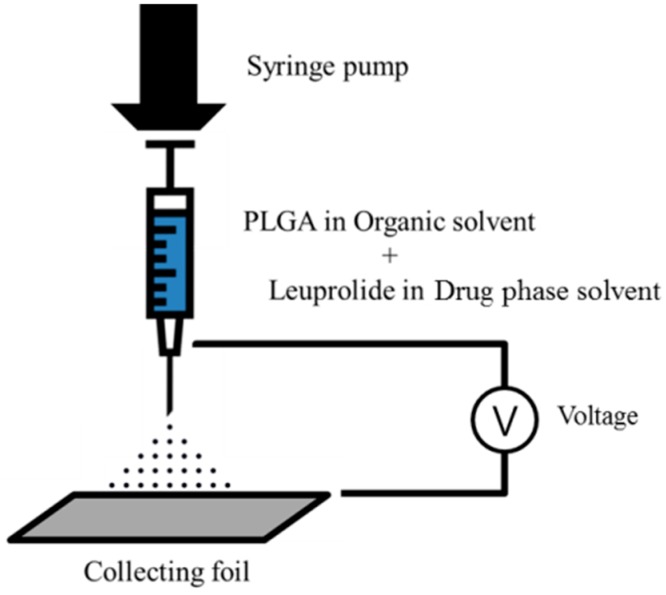
Schematic representation of the electrospraying procedure.

The formulation of electrosprayed microspheres was manufactured using ESR 100 electrospray equipment (NanoNC, Seoul, Korea). The PLGA and the leuprolide were dissolved in each solvent of polymer phase and drug phase, respectively, and the two solutions were completely mixed by vortexing for 5 min. These two mixed solutions were loaded in a 1 mL gas-tight syringe (Hamilton Co., Reno, NV, USA) equipped with a 23-gauge metal nozzle. The syringe was mounted on an electrospray device so that the distance between the tip of the nozzle and the collecting aluminum foil was 20 cm. Electrospraying was carried out under the following conditions: injection rate of 0.4 mL/h and an applied voltage of 16.1 to 18.6 kV. The dried matter was carefully obtained from collecting aluminum foil and stored in polyethylene tubes. The scheme of the electrospraying procedure is shown in Figure 1.

### 2.5. Optimization of Electrosprayed Microspheres

Solubility, viscosity, spray pattern, and density of polymer solution were investigated using four solvents (methylene chloride, acetonitrile, ethyl acetate, and acetone), and the optimal solvent was selected from among these. Parameters including dissolution of the leuprolide, miscibility with the solvent of polymer phase, drying degree of dry matter, and density of drug solution were investigated using three solvents (water, ethanol, and methanol), and the optimal solvent was selected. 

The degree of precipitation of PLGA according to the mixing ratio of the polymer solution and the drug solution was tested. Mixed solutions were prepared in which the ratios of the drug solution were 10%, 20%, 30%, 50%, 70%, 80%, and 90%, respectively.

After optimizing the solvent and the mixing ratio for two solutions, each of the trial formulations were prepared with various ratios of RG502 and RG502H. The structures of RG502 and RG502H are similar but differ in their end groups with RG502 and RG502H having ester and acid, respectively. Five types of microspheres were prepared with ratios of RG502 and RG502H of 100:0 (OL3), 75:25 (OL2), 50:50 (OL1), 25:75 (OL4), and 0:100 (OL5), respectively. All the variables are summarized in Table S1. 

### 2.6. Physicochemical Characterization of Electrosprayed Microspheres

#### 2.6.1. Scanning Electron Microscope (SEM)

Microsphere morphology was investigated by SEM (S-4800; Hitachi Ltd., Tokyo, Japan). First, the microspheres were affixed on metallic mount for coating and were coated with platinum using sputter coater (K575K; Emitech Ltd., Ashford, UK). After coating with platinum, the morphology of microspheres was observed at different zoom magnifications by SEM (S-4800; Hitachi Ltd., Tokyo, Japan). SEM images of microspheres after 1 and 28 days in release buffer were also measured.

#### 2.6.2. Differential Scanning Calorimetry (DSC)

The thermograms of electrosprayed microspheres, leuprolide powder, and PLGA including RG502 and RG502H were measured by DSC (DSC Q20; TA Instruments, New Castle, DE, USA). The nitrogen gas flowed at 25 mL/min as purge gas. The samples were heated from 20 to 200 °C at a heating rate of 10 °C/min.

#### 2.6.3. Fourier Transform Infrared Spectroscopy (FTIR)

To investigate the interaction between the drug and the polymers, FTIR spectrum by attenuated total reflectance method using an FTIR spectrophotometer-430 (Shimadzu Corporation, Tokyo, Japan) was performed. All samples containing leuprolide powder, PLGA, and leuprolide-loaded microspheres were scanned from 400 to 4000 cm^−1^ at a resolution of 2 cm^−1^.

### 2.7. Drug Content of Electrosprayed Microspheres

HPLC-UV was used for quantification of the leuprolide in microspheres. Each 3 mg of OL1 to 5 were added to 400 μL of water. In order to completely dissolve the microspheres, 1 mL of ethyl acetate was mixed and stirred for 3 min and then centrifuged at 10,000× *g* for 5 min. Thereafter, 200 μL of the aqueous phase was transferred to a clean tube, and then 50 μL was injected into the HPLC-UV system. The column was a Waters Nova-Pak^®^ C_18_ column (3.9 mm × 150 mm, 4 μm particle size, Waters Corporation, Milford, MA, USA), and the mobile phase composition was 1:1.5 ratio of 0.25 M ammonium acetate in water and methanol. The wavelength for UV detection was 280 nm, and the flow rate was 1.0 mL/min. 

### 2.8. In Vitro Release Test

The release test was conducted in a heating dry bath (confido–S20H, Seoul, Korea) at 300 rpm and 37 °C for 28 days using polyethylene tube for investigating release of the reference drug (Lucrin depot^®^) and trial formulations. Phosphate buffered saline (PBS, Mediatech, Inc., Manassas, VA, USA) containing 0.02% Tween 80 was used as the release media, and 10 mg of Lucrin depot^®^ or each trial formulation was added to 1 mL of release media to perform the release test. At predetermined sampling times (2 and 8 h, 1, 2, 4, 7, 10, 13, 18, 23, and 28 days), 0.8 mL of supernatant was collected after centrifugation at 3000× *g* for 3 min. Subsequently, 0.8 mL of fresh release media was added to the polyethylene tubes to maintain original volume. The concentration of the leuprolide in the collected supernatant was analyzed by HPLC-UV and quantified. 

### 2.9. In Vitro–In Vivo Correlation

#### 2.9.1. Development of IVIVC Model

**Figure 2:**
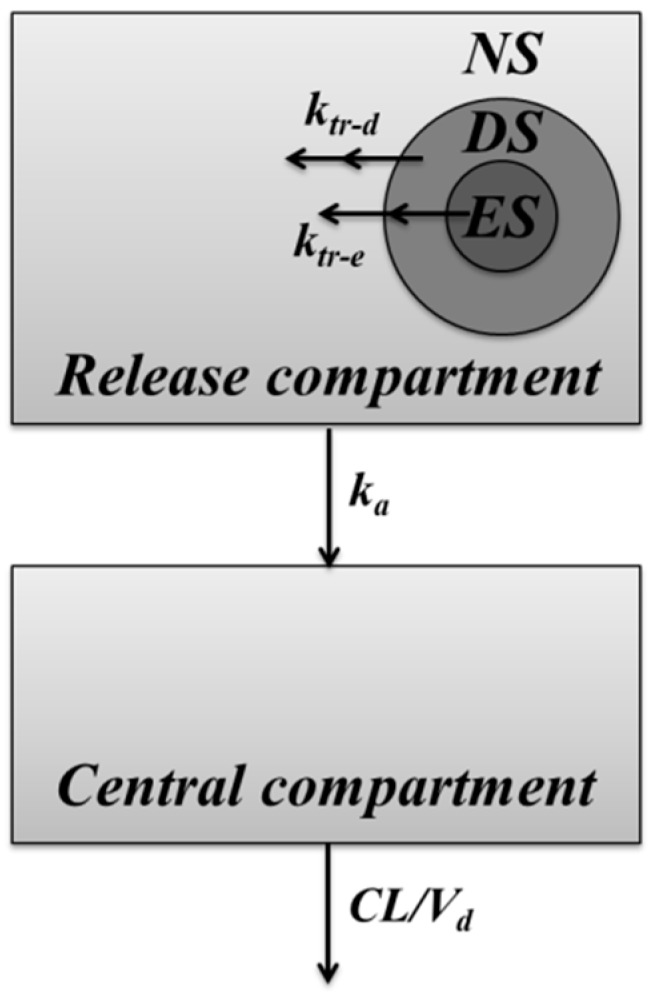
Schematic representation of the in vitro–in vivo correlation (IVIVC) model of microspheres. Solid lines indicate the elimination or the distribution of leuprolide.

The IVIVC model used in this study was modified from the PK model developed in our previous study of the leuprolide solution treated group and Lucrin depot^®^ treated group after SC administration [10]. The structure of developed IVIVC model is shown in Figure 2.

The IVIVC model development in this study was carried out using Berkeley Madonna (program version 8.3.14). Differences in PK profiles by formulations were evaluated by adding a release compartment to reflect the release characteristic. In order to express different release kinetics of the drug, the inside of the microsphere was divided into three virtual sections: the “non-capsuled release section (NS)”, which is not enclosed or contained on the surface of the microsphere, exhibits a PK profile similar to that of the leuprolide solution-administered group; “erosive-release section (ES)”, which is located at the deepest part of the formulation and releases the drug at a slow rate; and “diffusive release section (DS)”, which is released at a moderate rate between the two sections. k_tr-d_ is a diffusive release constant from DS to NS. k_tr-e_ is the erosive release constant from ES to NS. The ratios of drugs contained in each section of NS, DS, and ES were defined as E1, E2, and E3, respectively. E1 is the ratio of non-encapsulated drug that represents an initial value of the release compartment and reflects the initial burst release of the drug. The drug encapsulated in DS and ES can be expressed at various rates using a transit model. E2 and E3 are released into the release compartment by constants k_tr-d_ and k_tr-e_, respectively. Drugs in the release compartment are absorbed into the central compartment by the absorption rate constant (k_a_). Elimination of absorbed drugs was interpreted as clearance (CL) and volume of distribution (V_d_). k_a_, CL, and V_d_ were all estimated by one-compartmental analysis from the leuprolide solution-administered group of our previous study [10]. The value of PK parameters was 16.67 h^−1^ of k_a_, 514.46 mL/h of CL, 487.40 mL of V_d_, respectively. The equations for describing this IVIVC-PK model are as follows:

(1) $$\begin{array}{c} \frac{d\left(\mathrm{NS}\right)}{\mathrm{dt}}=k_{\mathrm{tr}-d}\cdot {\mathrm{DS}}_{n}+k_{\mathrm{tr}-e}\cdot {\mathrm{ES}}_{n}-k_{a}\cdot \mathrm{NS}\;\\ \frac{d({\mathrm{DS}}_{1})}{\mathrm{dt}}=-k_{\mathrm{tr}-d}\cdot {\mathrm{DS}}_{1}\;\\ \frac{d\left({\mathrm{DS}}_{n}\right)}{\mathrm{dt}}=k_{\mathrm{tr}-d}\cdot {\mathrm{DS}}_{n-1}-k_{\mathrm{tr}-d}\cdot {\mathrm{DS}}_{n}\\ \frac{d({\mathrm{ES}}_{1})}{\mathrm{dt}}=-k_{\mathrm{tr}-d}\cdot {\mathrm{ES}}_{1}\;\\ \frac{d\left({\mathrm{ES}}_{n}\right)}{\mathrm{dt}}=k_{\mathrm{tr}-d}\cdot {\mathrm{ES}}_{n-1}-k_{\mathrm{tr}-d}\cdot {\mathrm{ES}}_{n}\\ E1+E2+E3=1\\ \frac{d\left(\mathrm{Central}\right)}{\mathrm{dt}}=k_{a}\cdot \mathrm{NS}-\mathrm{CL}/V_{d}\cdot \mathrm{Central} \end{array}$$

where NS, DS_1_, DS_n_, ES_1_, ES_n_, and Central refer to the amount of leuprolide in each compartment. The IVIVC model estimates release parameters including E1, E2, E3, number of transit compartments of DS and ES, and rate constants including k_tr-d_ and k_tr-e_. Initial values of NS, DS_1_, and ES_1_ were dose·E1, dose·E2, and dose·E3, respectively.

#### 2.9.2. Correlation of In Vitro and In Vivo Release

Using the above modeling approach, E1, E2, E3, k_tr-d_ and k_tr-e_ were estimated by fitting the PK profile of Lucrin depot^®^-administered group to the IVIVC model. The release compartment consists of the release from the microspheres and absorption into the central compartment. By excluding the absorption of the drug into the central compartment, parameters of the release compartment can be applied to calculate the release rate of the drug from microspheres to the release compartment. In this case, the release of the drug from microspheres to the release compartment was defined as a predicted in vivo release. The predicted in vivo release rate can be regarded as the input rate that is absorbed into the central compartment. The correlation between in vitro dissolution and predicted in vivo dissolution was identified through nonlinear regression and was verified by using a levy plot.

#### 2.9.3. Prediction of PK Profiles from the In Vivo Dissolution

Using the correlation obtained through the regression analysis between in vitro release and predicted in vivo release, in vivo release profiles of five prepared microspheres can be predicted from the observed in vitro release profile. The predicted in vivo release profiles of each microsphere can be fitted to the developed IVIVC model, whereby the parameters of the release compartment were estimated. CL, V_d_, and k_a_ of the leuprolide solution-administered group were applied to the developed IVIVC model to predict the PK profiles of each formulation from the in vivo dissolution.

#### 2.9.4. Validation of the IVIVC Model

The established IVIVC model was validated according to FDA guidance (FDA, Guidance for industry: Extended release oral dosage forms, 1997). According to the guidance, the IVIVC should demonstrate the predictive performance of in vivo performance from its in vitro release characteristics. The FDA indicated that this verification of predictive performance can be confirmed by estimating the percent prediction error (%PE), which is calculated as follows:

(2) $$\%\mathrm{PE}=\frac{\left|\mathrm{Predicted}\;\mathrm{PK}\;\mathrm{Parameter}-\mathrm{Observed}\;\mathrm{PK}\;\mathrm{parameter}\right|}{\mathrm{Observed}\;\mathrm{PK}\;\mathrm{parameter}}\times 100$$

The FDA specifies that estimated %PE values of the maximum plasma concentration (C_max_) and the area-under-the-curve (AUC) in established IVIVC models should not exceed 15% for individual formulations and the average %PE values should not exceed 10%. This study validated the model established under these criteria.

### 2.10. Pharmacokinetic and Pharmacodynamic Study

#### 2.10.1. Experimental Design

Ten male rats were divided into two groups, the Lucrin depot^®^-administered group and the electrosprayed microspheres-administered group (*n* = 5/group). Either Lucrin depot^®^ or electrosprayed microspheres equivalent to 0.1 mg/kg of the leuprolide acetate were administered subcutaneously to each experimental group. Blood samples were directly drawn from the right jugular vein at predetermined time intervals (0, 0.25, 1, 2, 4, and 8 h, 1, 2, 3, 4, 5, 6, 7, 11, and 14 days). Approximately 300 μL of blood was collected in heparinized tubes via the catheter and was immediately centrifuged at 12,000× *g* for 5 min and then stored at −80 °C until analysis. 

#### 2.10.2. Bioanalysis of Rat Plasma

For PK or PD evaluation, quantification of the leuprolide or the testosterone from plasma samples was performed in the same way as previously [11]. A validated method was used to quantify the leuprolide in plasma and evaluate the PKs. The plasma concentration of testosterone was quantified to evaluate the PDs of the leuprolide using testosterone ELISA kits (R&D Systems, Minneapolis, MN, USA). 

### 2.11. PK-PD Evaluation and Data Analysis

The PK analysis in both groups was performed by non-compartmental analysis using WinNonlin^®^ software (version 8.1, Certara USA, Inc., Princeton, NJ, USA). The C_max_ was obtained from the observation of the individual plasma concentration–time curve. The AUC_inf_ was measured by linear trapezoidal rule from time zero to the last measured concentration and was extrapolated from the last measured concentration to infinity. The CL/F was calculated as the dose of leuprolide divided by AUC_inf_. All data were tested for statistical significance by the Mann–Whitney U test with *p* < 0.05 indicating a significant difference.

## 3. Results and Discussion

### 3.1. Optimization of Electrosprayed Microspheres

For preparation of electrosprayed microspheres, the solutions containing drug and polymer should have a clearly dissolved state, low viscosity, and high miscibility between solvents of each drug and polymer phase. Therefore, we optimized each solvent of the drug phase and the polymer phase for obtaining optimal microspheres. First, methylene chloride, acetonitrile, ethyl acetate, and acetone were compared as solvent candidates for the polymer phase. Methylene chloride has been the most frequently used as a solvent for PLGA. It showed high solubility for PLGA, relatively low viscosity among solvents, and formed a uniform spray pattern. However, according to the International Council for Harmonization of Technical Requirements for Pharmaceuticals for Human Use (ICH) Q3C guideline, methylene chloride is classified as a class 2 solvent that should be limited in pharmaceutical products because of its inherent toxicity. Also, methylene chloride has a density of 1.326 g/cm^3^, which is not similar to the density of the solvent for the drug phase. Acetonitrile and ethyl acetate showed relatively high viscosity, and their spray pattern was irregular. Ultimately, the polymer phase solvent chosen was acetone. Acetone is classified as a class three solvent that may be regarded as less toxic and of lower risk to human health. Also, it exhibited high solubility for PLGA and had relatively low viscosity. The density of acetone is 0.785 g/cm^3^, which is similar to the water or the methanol used as the solvent of the drug phase. Because of these properties, acetone has high miscibility with the solvent of the drug phase and a uniform spray pattern can be formed. The test results for selection of the polymer phase solvent are summarized in Table S2.

Secondly, ethanol, water, and methanol were compared as solvent candidates for the drug phase. Ethanol dissolves leuprolide slowly, and the drug easily precipitated. Water showed high solubility for the leuprolide and good miscibility with acetone (the solvent of the polymer phase), but it was difficult to obtain electrosprayed microspheres because drying degree of dry matter was incomplete. Methanol was finally chosen as the solvent for the drug phase and showed high solubility for the leuprolide and good miscibility with acetone. In addition, the dry matter was completely dried, and it was easy to collect electrosprayed microspheres. The test results for selection of the drug phase solvent are summarized in Table S3.

Lastly, the mixing ratio of polymer solution and drug solution was investigated. The leuprolide and the PLGA were dissolved in methanol and acetone, respectively, and the two solutions were mixed. When the ratios of the drug solution were 10%, 20%, and 30%, respectively, the appearance of the mixed solutions was all transparent. When the ratios of the drug solution were 50% and 70%, respectively, the appearance became cloudy. When the ratios of the drug solution were 80% and 90%, respectively, PLGA precipitated from the mixed solution. This suggests that the solubility of the leuprolide and the PLGA depends on the ratio of methanol to acetone. The composition of the solvent mixture was finally determined to be acetone with methanol at 80 to 20. The test results for the mixing ratio are shown in Table S4.

### 3.2. Physicochemical Characterization of Electrosprayed Microspheres

**Figure 3:**
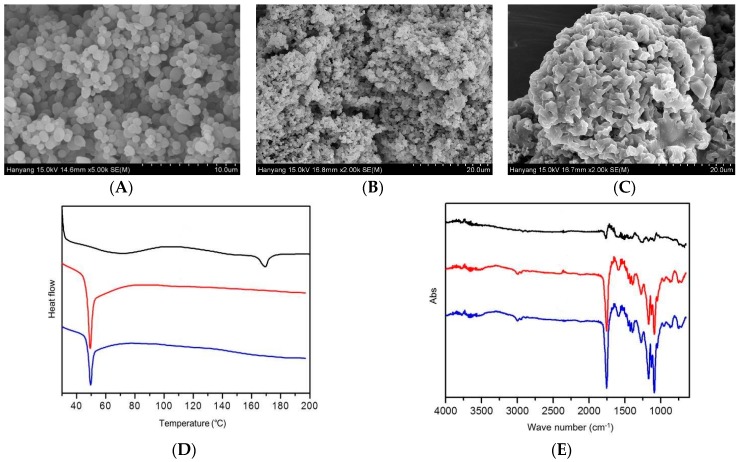
Physicochemical properties of electrosprayed microspheres. Scanning electron micrographs of electrosprayed microspheres (**A**) and after one day (**B**) and 28 days (**C**) in release buffer. Differential scanning calorimetry thermograms of leuprolide (black line), poly (d,l-lactide-co-glycolide) (PLGA) (red line), and electrosprayed microspheres (blue line) (**D**). Fourier transform infrared spectroscopy spectra of leuprolide (black line), PLGA (red line), and electrosprayed microspheres (blue line) (**E**).

SEM images show the morphology of electrosprayed microspheres. As shown in Figure 3A, the particle size of the microspheres obtained by electrospraying was distributed in the range of 6.0 to 11.0 μm and was in the shape of an ellipse or appeared as globular. The SEM images of microspheres after one and 28 days in release buffer are shown in Figure 3B,C. As the microspheres were left in the release buffer for a long time, the surface of the microsphere exhibited aggregation or degradation. On the last day of the release test, most of the microspheres were clustered, and the surface of the microspheres collapsed. Only a small number of particles still retained their shape.

The DSC thermograms of leuprolide, PLGA, and electrosprayed microspheres are illustrated in Figure 3D. From the DSC pattern, leuprolide showed an endothermic peak at about 171 °C, which seemed to be due to a leuprolide phase change. PLGA showed an endothermic peak at around 49 °C, and the same pattern also appeared in electrosprayed microspheres. In addition, no leuprolide peak appeared in the thermogram of electrosprayed microspheres, suggesting that the leuprolide was well encapsulated in the microsphere during electrospraying.

Figure 3E shows the FTIR spectra of leuprolide, PLGA, and electrosprayed microspheres. No distinct peaks were observed in the FTIR spectrum of leuprolide. In the PLGA spectrum, peaks were observed at 746.45, 867.97, 1089.78, 1168.86, and 1755.22 cm^−1^. These peaks were also observed in electrosprayed microspheres, confirming that no additional covalent bonds were formed during electrospraying.

### 3.3. Drug Content of Electrosprayed Microspheres

The mean percent of drug content in each electrosprayed microsphere preparation was investigated (*n* = 3). The proportion of leuprolide was over 90% in all trial formulations. The leuprolide content was 99.37% for OL1, 94.73% for OL2, 98.67% for OL3, 93.49% for OL4, and 93.75% for OL5. 

### 3.4. In Vitro Release Test

**Figure 4:**
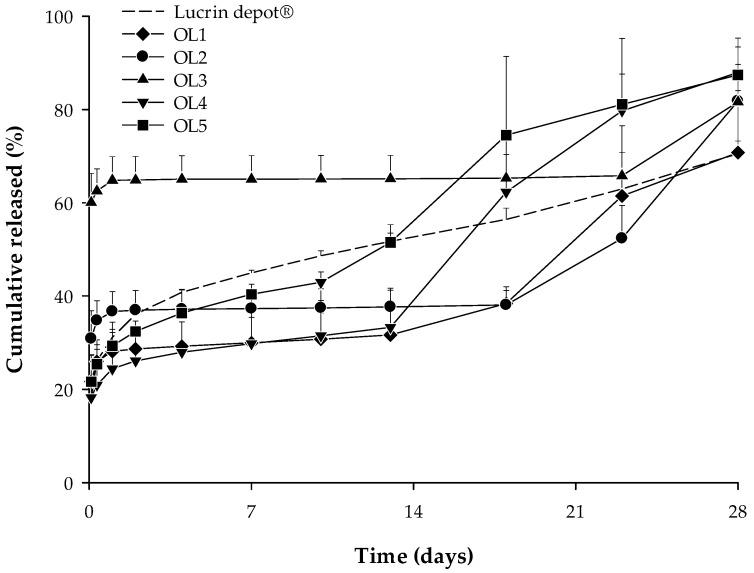
In vitro release profile for Lucrin depot^®^ and each trial formulation (OL1, OL2, OL3, OL4, and OL5) (mean ± SE, *n* = 3).

In vitro release tests were performed on five trial formulations (OL1, OL2, OL3, OL4, and OL5) and the reference drug (Lucrin depot^®^) for 28 days (mean ± SE, *n* = 3). The results show over 60% of the leuprolide was released in all trial formulations until day 28, as shown in Figure 4. 

Of all the trial formulations in which the release test was performed, one was selected for the PK study. 

### 3.5. In Vitro–In Vivo Correlations

#### 3.5.1. Correlation of In Vitro and In Vivo Release

In order to obtain the predicted in vivo release profile, parameters of the release compartment were first estimated by fitting the PK profile of Lucrin depot^®^ to the IVIVC model. The estimated parameters were 0.190 for E1, 0.400 for E2, 0.410 for E3, 1.000 for number of transit (DS), 2.000 for number of transit (ES), 0.080 h^−1^ for k_tr-d_, and 0.008 h^−1^ for k_tr-e_, respectively.

In the release compartment represented by estimated parameters, the in vivo release profile was predicted by excluding the drug absorbed into the central compartment. 

**Figure 5:**
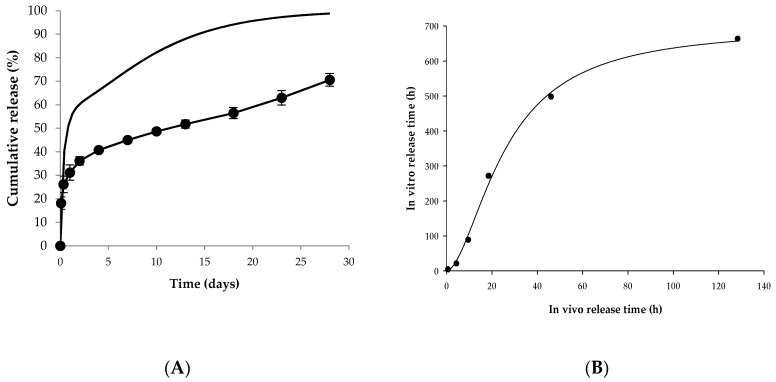
In vitro–in vivo correlation. (**A**) Observed in vitro release profile and predicted in vivo release profile of Lucrin depot^®^. Closed circles mean the observed in vitro release profile. Solid line indicates the predicted in vivo release profile. (**B**) The correlation between times representing the same percent of cumulative release in vitro and in vivo.

As shown in Figure 5A, the predicted in vivo release rate was much faster than the in vitro release rate. In general, sustained release drugs modeled by IVIVC tend to have a faster release rate in vitro than in vivo, but PLGA microspheres tend to have a faster release rate in vivo [12]. Similarly, the in vivo release rate seen in this study was faster than in vitro release rates. 

The correlation between times indicating the same cumulative release (%) in vitro and in vivo was verified. Table 1 shows in vivo and in vitro times according to the percentage of cumulative release. The correlation was calculated between in vitro and in vivo times in which percentages of cumulative release reached 20%, 30%, 40%, 50%, 60%, and 70%, respectively. The cumulative release of the first sampling point of the in vitro release test was about 18%, thus the time at 10% cumulative release was not correlated. Also, since the cumulative release of the last sampling point was about 70%, indicating incomplete drug release appeared, the subsequent time was excluded from the correlation. Therefore, only six time points were correlated with cumulative releases of 20%, 30%, 40%, 50%, 60%, and 70% due to the rapid release at the early stage and the incomplete drug release.

The correlation was verified by time scaling using the Levy plot and the Hill equation. The Hill equation constants were estimated by nonlinear regression. The Levy plot, designed by Levy in 1967, is one of the most traditional time scaling tools. This graph plots the times representing the same or a similar percent of cumulative release in vivo and in vitro. This method of time scaling can bridge the time gap between in vitro and in vivo release. The result of the Levy plot is depicted in Figure 5B, and the Hill equation for the correlation is described below:

(3) $$\begin{array}{c} y=\frac{a\times x^{b}}{c^{b}+x^{b}}\\ \mathrm{in}\;\mathrm{vitro}\;\mathrm{release}\;\mathrm{time}=\frac{696.87\times {\left(\mathrm{in}\;\mathrm{vivo}\;\mathrm{release}\;\mathrm{time}\right)}^{1.75}}{25.91^{1.75}+{\left(\mathrm{in}\;\mathrm{vivo}\;\mathrm{release}\;\mathrm{time}\right)}^{1.75}} \end{array}$$

The regression analysis yielded three Hill equation constants: 696.87 for *a*, 1.75 for *b*, and 25.91 for *c* with the R-square of 0.997. 

#### 3.5.2. Prediction of PK Profiles from the In Vivo Dissolution

In vivo release profiles of OLs were predicted from in vitro release profiles using time correlation. In vitro release profiles and predicted in vivo release profiles of each formulation are shown in Figure S1. By fitting the predicted in vivo release profile to the release compartment of the IVIVC model that excluded absorption, the parameters of the release compartment were estimated and are shown in Table 2. The estimated E1, E2, E3, number of transit compartment of DS, number of transit compartment of ES, k_tr-d_, and k_tr-e_ were 0.173, 0.533, 0.294, 2.478, 2.210, 0.049 h^−1^, and 0.001 h^−1^ for OL1, respectively, and 0.300, 0.578, 0.122, 2.364, 2.000, 0.026 h^−1^, and 0. 00006 h^−1^ for OL2, respectively, and 0.610, 0.086, 0.304, 2.759, 2.195, 0.045 h^−1^, and 0.010 h^−1^ for OL3, respectively, and 0.210, 0.780, 0.010, 2.020, 2.068, 0.047 h^−1^, and 0.0000002 h^−1^ for OL4, respectively, and 0.093, 0.791, 0.116, 2.002, 2.000, 0.093 h^−1^, and 0.009 h^−1^ for OL5, respectively.

**Figure 6:**
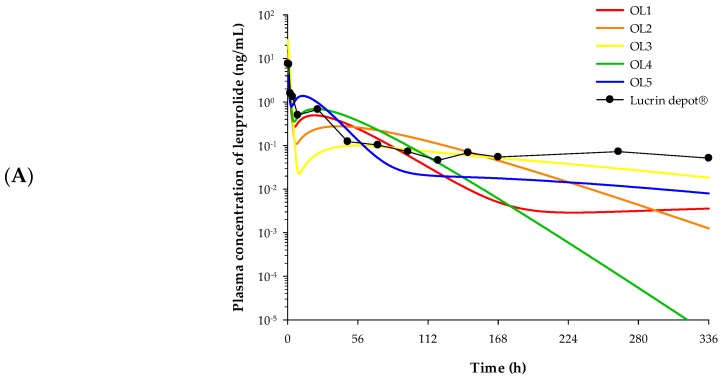

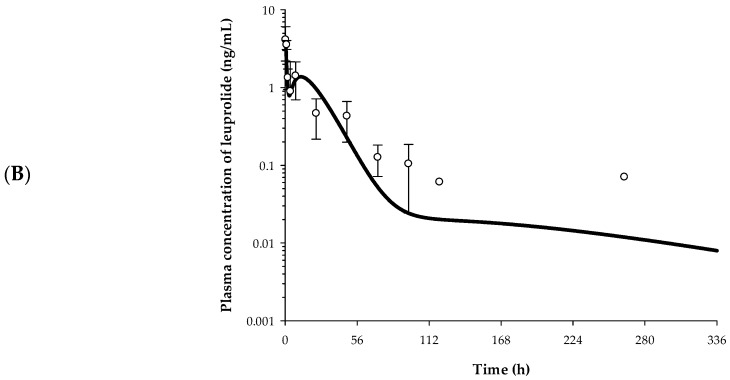
Plasma concentration–time profile described by the IVIVC-PK (pharmacokinetic) model. (**A**) The observed PK profile of Lucrin depot^®^ and the predicted PK profiles of five electrosprayed microsphere preparations. Closed circles refer to observational value after Lucrin depot^Ⓡ^ administration. (**B**) The observed and the predicted PK profiles of OL5 (*n* = 5). Open circles refer to the observed plasma concentration of leuprolide. Solid line indicates the model predicted PK profile.

The PK profiles of each formulation were predicted by applying the estimated parameters of the release compartment and the PK parameters of the leuprolide solution-administered group to the developed IVIVC model. The observed PK profile of Lucrin depot^®^ and the predicted PK profiles of five electrosprayed microspheres are depicted in Figure 6A.

The PK parameters of each formulation were estimated via non-compartmental analysis (NCA) based on the predicted PK profiles and are noted in Table 3. The C_max_ of OL1, OL2, OL3, OL4, and OL5 were 7.46, 12.93, 26.29, 9.05, and 4.01 ng/mL, respectively. The AUC_inf_ of OL1, OL2, OL3, OL4, and OL5 were 36.82, 46.10, 54.21, 51.20, and 52.52 h·ng/mL, respectively. The CL/F of OL1, OL2, OL3, OL4, and OL5 were 679.03, 542.29, 461.21, 488.28, and 475.97 mL/h, respectively.

The PK profiles and the PK parameters of the five formulations obtained from the developed IVIVC model were used to select the optimal formulation without performing actual in vivo PK studies. Of the five formulations, OL1 was predicted to have lower AUC_inf_ than Lucrin depot^®^. This is inferred to result in rapid elimination of the drug due to high clearance and may not be as effective as desired due to insufficient in vivo exposure. For OL2 and OL3, an excessively high C_max_ was predicted, which may cause burst release of the leuprolide from microspheres and a subsequent sharp rise in testosterone. Burst release is a phenomenon seen in many controlled release formulations and can result in local or systemic toxicity from high concentrations of drugs as well as reduced half-life in vivo [13]. Although OL4 was the most similar formulation to Lucrin depot^®^ in terms of PK parameters, OL5 with a lower C_max_ than OL4 was chosen as the optimal formulation. The lower C_max_ compared to Lucrin depot^®^ probably indicates that burst release of the leuprolide from microspheres and “testosterone flare” could be reduced. The testosterone flare is a temporary increase in testosterone levels in the body caused by certain types of hormone therapy used to treat prostate cancer (National Cancer Institute). In addition, the AUC_inf_ of OL5 was the most similar to Lucrin depot^®^ and showed a slow release forming a plateau in the elimination phase. For these reasons, OL5 was selected as the final formulation among the five microsphere candidates.

#### 3.5.3. Validation of the IVIVC Model

The IVIVC model was validated by the in vivo PK study of OL5. The PK profile of OL5 was estimated from actual in vivo experiments, and %PE values were evaluated by comparing with the predicted PK profile. %PE values were evaluated for C_max_ and AUC_inf_. Each PK parameter was estimated using NCA. In OL5, the predicted and the observed values of C_max_ were 4.01 and 4.14 ng/mL, respectively. The predicted and the observed values of AUC_inf_ were 52.52 and 56.95 h·ng/mL, respectively. %PE of C_max_ and AUC_inf_ were 3.14% and 7.78%, respectively. All four parameters showed %PE values within 10% and were calculated with an average of 5.46%. The developed IVIVC model therefore satisfies the validation criteria of the FDA, which states that that the individual PE should not exceed 15% and the average PE should not exceed 10%. The observed and the predicted PK profile of OL5 are shown in Figure 6B.

### 3.6. Pharmacodynamic Evaluation

**Figure 7:**
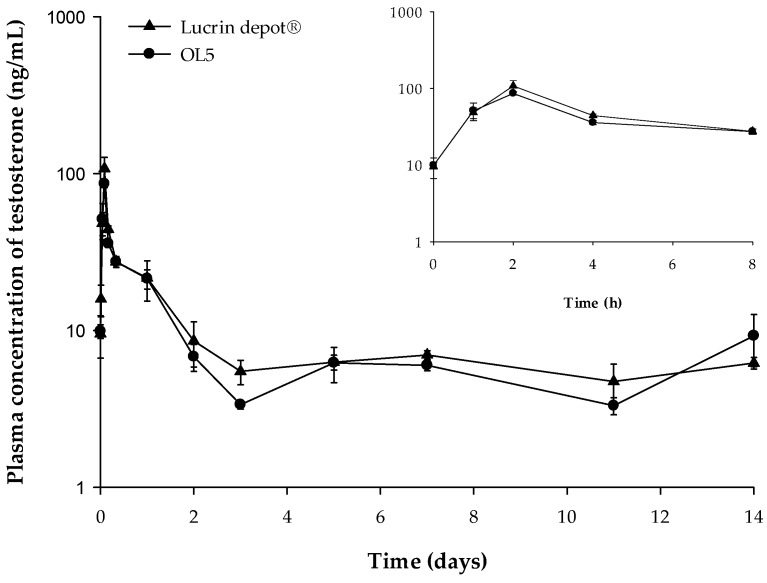
The pharmacodynamic profiles after subcutaneous (SC) administration of Lucrin depot^®^ or OL5 in rats. The inset shows the short-term pharmacodynamic (PD) profile up to 8 h (mean ± SE, *n* = 5).

PD study of electrosprayed microspheres (OL5) containing leuprolide was carried out for evaluating the suppressive effects of testosterone level. The plasma concentration–time curve of testosterone after SC administration of Lucrin depot^®^ and OL5 is shown in Figure 7. 

The C_max_ of testosterone of the Lucrin depot^®^-administered group and the OL5-administered group were 107.8, and 86.2 ng/mL, respectively. The C_max_ of the leuprolide was significantly lower in OL5 than Lucrin depot^®^. However, the C_max_ of testosterone in OL5 was not significantly different from Lucrin depot^®^. In addition, the mean concentrations of testosterone from three to 14 days were 5.93 and 5.63 ng/mL in the Lucrin depot^®^ and OL5, respectively, with no significant difference. The PD evaluation suggests that the efficacy of OL5 is similar to Lucrin depot^®^, but the formulation was improved by reducing the initial burst release. 

## 4. Conclusions

In this study, leuprolide and PLGA were formulated into microspheres via the electrospray method. The content of leuprolide in microspheres was calculated, and the in vitro dissolution test was performed. In the IVIVC model, parameters of the release compartment were first estimated by fitting the PK profile of Lucrin depot^®^. In the release compartment, the in vivo release profile of Lucrin depot^®^ was predicted by excluding the drug absorbed into the central compartment. The correlation between times indicating the same cumulative release (%) in vitro and in vivo was verified by time scaling. Also, in vivo release profiles of trial formulations were predicted from in vitro release profiles using the time correlation. The parameters of each formulation in release compartments were estimated by fitting the in vivo release profiles to the IVIVC model that excluded absorption. Finally, applying estimated parameters of the release compartment and the PK parameters to the developed IVIVC model, the PK profiles of OLs were predicted. Using the predicted PK profiles, OL5 was selected as the optimal formulation, as it represented similar AUC_inf_ and a lower C_max_ compared to Lucrin depot^®^. The PK and the PD profiles after SC administration of OL5 were evaluated in rats. As predicted using the developed IVIVC model, the AUC_inf_ was not significantly different from the Lucrin depot^®^, and the C_max_ was significantly decreased. These results indicate that the IVIVC model is well developed and validated to predict the PK profile from the in vitro dissolution. The lower C_max_ of leuprolide in OL5 compared to Lucrin depot^®^ probably indicates that the burst release could be reduced. However, the C_max_ of testosterone in OL5 was similar to Lucrin depot^®^. This suggests that the efficacy is similar to Lucrin depot^®^, but the formulation was improved by reducing the initial burst release.

## 5. Patents

The method for manufacturing SR microsphere containing leuprolide was patented under application No. 10-2017-0108842 (2017.08.28, Republic of Korea) and under the patent name “Method for manufacturing sustained release microsphere containing leuprolide prepared by electrospraying”.

## Figures and Tables

**Table 1:** In vivo and in vitro time according to the percentage of cumulative release.

Cumulative Release (%)	In Vivo Time (h)	In Vitro Time (h)
20	0.7	3.4
30	4.4	20.4
40	9.6	88.2
50	18.6	270.9
60	46.2	497.3
70	128.5	663.0

**Table 2:** Estimated parameters of the release compartment obtained by fitting the predicted in vivo release profile of electrosprayed microspheres.

Parameters	Estimates					
	OL1	OL2	OL3	OL4	OL5	Lucrin Depot^®^
E1	0.173	0.300	0.610	0.210	0.093	0.190
E2	0.533	0.578	0.086	0.780	0.791	0.400
E3	0.294	0.122	0.304	0.010	0.116	0.410
Number of transit (DS)	2.478	2.364	2.759	2.020	2.002	1.000
Number of transit (ES)	2.210	2.000	2.195	2.068	2	2.000
k_tr-d_ (h^−1^)	0.049	0.026	0.045	0.047	0.093	0.080
k_tr-e_ (h^−1^)	0.001	0.00006	0.010	0.0000002	0.009	0.008

DS: diffusive release section; ES: erosive release section.

**Table 3:** Estimated PK parameters from the observed PK profile of Lucrin depot^®^ and the IVIVC-PK model predicted PK profiles of five electrosprayed microspheres.

Parameters	OL1	OL2	OL3	OL4	OL5	Lucrin Depot^®^
C_max_ (ng/mL)	7.46	12.93	26.29	9.05	4.01	8.53
AUC_inf_ (h·ng/mL)	36.82	46.10	54.21	51.20	52.52	52.51

AUC: area-under-the-curve.

## Supplementary Materials

The following are available online at https://www.mdpi.com/1999-4923/12/1/36/s1, Figure S1: The observed in vitro release profiles and the predicted in vivo release profiles of OL1 (A), OL2 (B), OL3 (C), OL4 (D), and OL5 (E), Table S1: Composition of trial formulations and conditions of electrospraying, Table S2: Test results of solvents for the polymer phase, Table S3: Test results of solvents for the drug phase, Table S4: Mixing ratio of polymer and drug solution.
