# Supplementary material for: Development of Level A In Vitro–Vivo Correlation for Electrosprayed Microspheres Containing Leuprolide: Physicochemical, Pharmacokinetic, and Pharmacodynamic Evaluation
Source: Pharmaceutics. 2020 Jan 2;12(1):36. doi: 10.3390/pharmaceutics12010036 (PMC7022573; doi:10.3390/pharmaceutics12010036)
Supplement: Supplementary file 1 [file pharmaceutics-12-00036-s001.pdf]

pharmaceutics

Supplementary Materials: Development of Level A In Vitro-In Vivo Correlation for Electrosprayed Microspheres Containing Leuprolide: Physicochemical, Pharmacokinetic, and Pharmacodynamic Evaluation

Dong-Seok Lee, Dong Wook Kang, Go-Wun Choi, Han-Gon Choi, and Hea-Young Cho

Table S1. Composition of trial formulations and conditions of electrospraying.

Trial Formulation	Polymer (mg)	Polymer Solvent (mL)	Drug (mg)	Drug Solvent (mL)	Voltage (kV)
OL1	RG502 50<br>RG502H 50	0.8	5	0.2	16.1
OL2	RG502 75<br>RG502H 25	0.8	5	0.2	16.1
OL3	RG502 100<br>RG502H 0	0.8	5	0.2	17.4
OL4	RG502 25<br>RG502H 75	0.8	5	0.2	16.1
OL5	RG502 0<br>RG502H 100	0.8	5	0.2	18.6

Table S2. Test results of solvents for the polymer phase.

Solvent	Dissolution of PLGA	Viscosity	Spray Pattern	Density (g/cm^3)
Methylene chloride	O	low	uniform	1.326
Acetonitrile	O	high	irregular	0.786
Ethyl acetate	O	high	irregular	0.895
Acetone	O	low	uniform	0.785

Table S3. Test results of solvents for the drug phase.

Solvent	Dissolution of Leuprolide	Miscibility with Acetone	Drying Degree of Dry Matter	Density (g/cm^3)
Ethanol	X	O	incomplete	0.789
Water	O	O	incomplete	0.998
Methanol	O	O	complete	0.791

Table S4. Mixing ratio of polymer and drug solution.

Ratio of Drug Solution (%)	Appearance of Mixed Solution
10	transparent
20	transparent
30	transparent
50	cloudy
70	cloudy
80	precipitated
90	precipitated

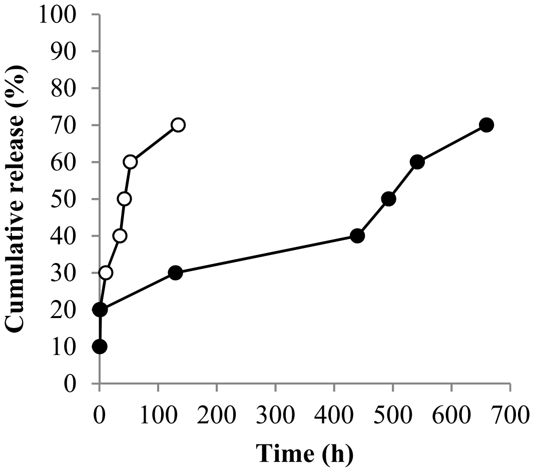

(**A**)

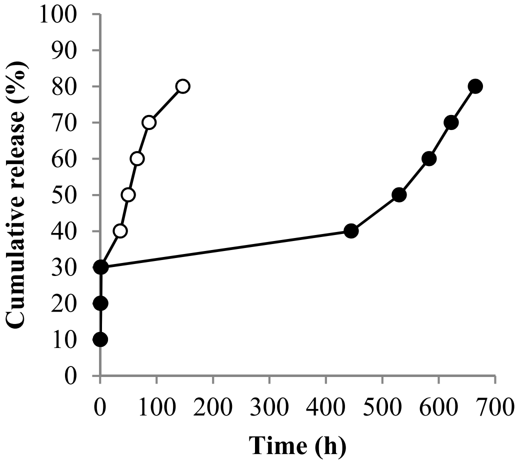

(**B**)

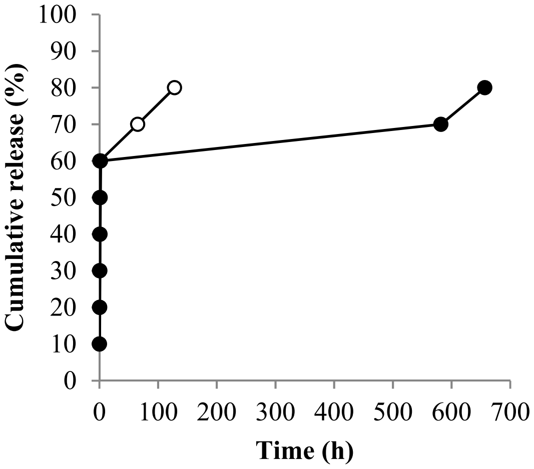

(**C**)

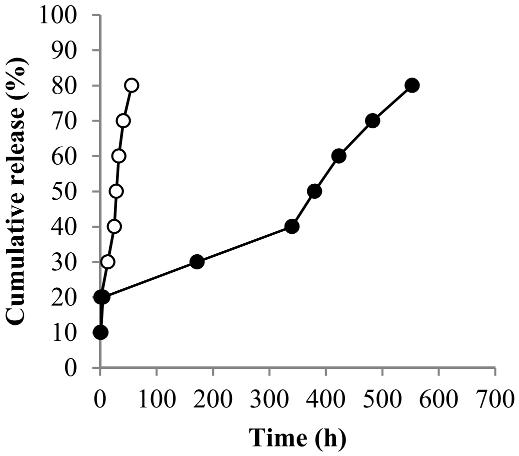

(**D**)

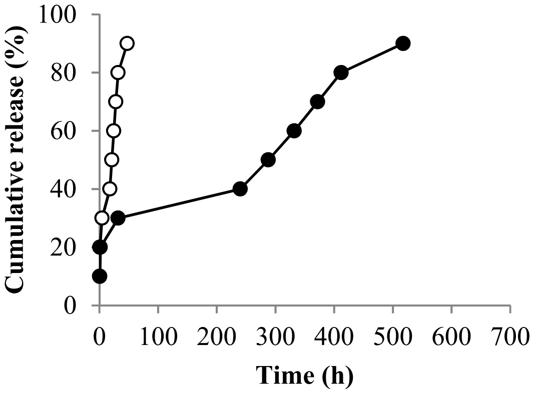

(**E**)

Figure S1. The observed in vitro release profiles and the predicted in vivo release profiles of OL1 (**A**), OL2 (**B**), OL3 (**C**), OL4 (**D**), and OL5 (**E**). Closed circles indicate the observed in vitro release profile. Open circles indicate the predicted in vivo release profile.
